# How does white matter microstructure differ between the vascular and amnestic mild cognitive impairment?

**DOI:** 10.18632/oncotarget.13960

**Published:** 2016-12-15

**Authors:** Yang Yu, Xinyu Liang, Haikuo Yu, Weina Zhao, Yan Lu, Yue Huang, Changhao Yin, Gaolang Gong, Ying Han

**Affiliations:** ^1^ Department of Neurology, Hongqi Hospital of Mudanjiang Medical University, Mudanjiang, Heilongjiang, China; ^2^ Department of Neurology, XuanWu Hospital of Capital Medical University, Beijing, China; ^3^ State Key Laboratory of Cognitive Neuroscience and Learning & IDG/McGovern Institute for Brain Research, Beijing Normal University, Beijing, China; ^4^ Center of Alzheimer's Disease, Beijing Institute for Brain Disorders, China; ^5^ Department of Rehabilitation, XuanWu Hospital of Capital Medical University, Beijing, China; ^6^ Department of Ophthalmology, Xuan Wu Hospital, Capital Medical University, Beijing, China; ^7^ School of Medical Sciences, Faculty of Medicine, UNSW Australia, Sydney, Australia

**Keywords:** mild cognitive impairment, neuroimage, Gerotarget

## Abstract

Changes in white matter (WM) microstructure may relate to the pathophysiology of cognitive impairment. Whether WM microstructure differs in two common pre-dementia subtypes, vascular mild cognitive impairment (VaMCI) and amnestic mild cognitive impairment (aMCI), is largely unknown. This study included 28 VaMCI (12 men, age: 46 ~ 77 years) and 34 aMCI patients (14 men, age: 51 ~ 79 years). All patients underwent a battery of neuropsychological tests and structural and diffusion magnetic resonance imaging (MRI) scanning. WM microstructure was quantified using diffusion MRI parameters: fractional anisotropy (FA), mean diffusivity (MD), axial diffusivity (AxD) and radial diffusivity (RD). These parameters were compared between the two patient groups using tract-based spatial statistics (TBSS) after controlling for age, gender, and education. No significant differences in FA/MD/AxD/RD were observed between the VaMCI and aMCI groups, which suggests a similar pattern of WM microstructure in the early stage of cognitive impairment for different dementia types. However, the two groups exhibited significant differences in the relationship between FA and the Auditory Verbal Learning Test (AVLT), which were primarily located around the corona radiate and corpus callosum. Specifically, there were significant positive correlations (R = 0.64, *P* < 0.001) between the FA and AVLT in the VaMCI group, but the opposite trend was observed in the aMCI group (R = −0.34, *P* = 0.047). The differential relationship between WM and memory between VaMCI and aMCI indicates an independent neuropathology for specific memory deficits in different types of dementia.

## INTRODUCTION

Vascular cognitive disorders (VCD) represent a wide spectrum of cognitive disorders associated with vascular causes, including vascular mild cognitive impairment (VaMCI) and vascular dementia (VD). VaMCI is defined as the prodromal stage of VD [1, 2]. Another common mild cognitive impairment (MCI) is amnestic MCI (aMCI), which represents the prodromal stage of Alzheimer's disease (AD) [3]. VaMCI and aMCI are highly prevalent MCI subtypes, and both conditions are associated with deficits in multiple cognitive domains, with identical chief complaints in memory deficits [4, 5]. Previous studies suggested that VaMCI patients exhibited more impairment in semantic memory and executive function, and deficits in episodic memory primarily characterized aMCI patients[6, 7].

White matter (WM) plays a critical role in normal cognitive functioning. Aberrant WM microstructure is observed in aMCI and aMCI [8–12]. Notably, VD and AD exhibit different WM impairments in transcallosal prefrontal tracts [13, 14]. However, no direct neuroimaging comparisons between VaMCI and aMCI were performed. Therefore, whether differences in WM impairments appear at very early stages of these two main dementia subtypes is not known.

The present study examined whether WM microstructure differed between VaMCI and aMCI by directly comparing two groups of age-matched patients and whether the relationships between WM and cognition differed between the two groups using diffusion magnetic resonance imaging (MRI) data combined with the tract-based spatial statistics (TBSS) method.

## RESULTS

### Demography and neuropsychological tests

Table 1 shows that no significant differences were observed for age, gender, or education between the two groups. There was a strong difference between the Auditory Verbal Learning Test (AVLT) delayed recall (AVLT_D_) and delayed recognition (AVLT_R_) scores between the two groups (*P* < 0.01), but no differences were found in the Mini-Mental State Examination (MMSE), Montreal Cognitive Assessment (MoCA) and AVLT immediate recall (AVLT_I_) scores. AVLT_D_ and AVLT_R_ were higher in the VaMCI group than the aMCI group. WM hyperintensity (WMH)/lacunar infarcts were observed in 52 of the 62 subjects. The aMCI group exhibited significantly lower Fazekas scale (FS) scores compared to the VaMCI group (*P* < 0.01) (Table 1). The spatial distribution of abnormal regions for the two groups was not significantly different (χ^2^ = 12.17, df = 10, *P* = 0.27).

**Table 1:** Demographic information and cognitive testing of the cohorts

	aMCI (*n*= 34)	VaMCI (*n*= 28)	Group Comparison (*p* value)
**Gender (Male/Female)**	14/20	12/16	0.894
**Age (yrs)**	65.50±8.48	63.75±9.23	0.440
**Education (yrs)**	10.21±4.18	8.71±3.68	0.146
**Auditory Verbal Learning Test (AVLT)**			
*Immediately recall*	6.21±1.59(34)	6.42±1.69(28)	0.616
*Delayed recall*	3.65±3.30(34)	6.46±2.89(28)	0.001^**^
*Delayed recognition*	7.18±4.66(34)	10.29±2.21(28)	0.002^**^
**MMSE**	24.38±3.89(34)	24.57±5.71(27)	0.878
**MoCA**	19.79±4.57(34)	17.46±7.36(25)	0.133
**Fazekas score**	0.91±0.75(34)	2.54±1.35(28)	0.000^**^

** Indicates *p* < 0.01

MMSE, the Mini-Mental State Examination; MoCA, the Montreal Cognitive Assessment. Chi-squared test for gender and Student's t tests (two-sided) for the remaining indices were performed to detect differences between the two groups.

### The association between neuropsychological performance and FS score

There was no significant FS × group interaction effect for any of the neuropsychological scores, which suggests that the relationship between cognitive performance and the FS score did not differ between the two groups.

### Group comparison of diffusion MRI parameters

There were no significant differences between the VaMCI and aMCI groups in any of the diffusion MRI parameters, i.e., fractional anisotropy (FA), mean diffusivity (MD), axial diffusivity (AxD) and radial diffusivity (RD).

### The association between diffusion MRI parameters and neuropsychological performance

**Figure 1:**
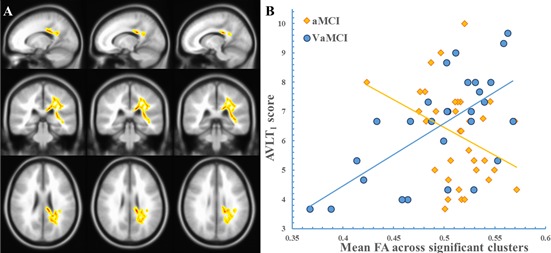
The “FA × Group” interaction effect on the AVLT_L_ The clusters showing a significant “FA × group” effect are indicated in a yellow-to-red color. The color represents the F statistic for this interaction. The scatter plot was drawn using the average FA value of the cluster. Panel **A**, region with a significant “FA × group” effect. Panel **B**, the scatterplot.

Figure 1 shows that multiple clusters exhibited significant “FA × group” interactions on the AVLT_I_. The significant WM clusters [family-wise error (FWE)-corrected *P* < 0.05] were primarily located around the following areas: (1) projection fibers involving the left posterior corona radiata (PCR), superior corona radiate (SCR), posterior thalamic radiation, and internal capsule; (2) association fibers involving the left sagittal stratum (including the inferior longitudinal fasciculus and inferior fronto-occipital fasciculus), superior longitudinal fasciculus (SLF); and (3) the splenium and body of the corpus callosum (CC). Figure 1 displays the scatterplot in which the mean FA across all significant clusters was used. Post hoc analysis revealed a positive correlation (R = 0.64, *P* < 0.001) between the FA and AVLT_I_ score in the VaMCI group but a negative correlation in the aMCI group (R = −0.34, *P* = 0.047). Notably, the scatterplot of each cluster revealed a very similar pattern for the relationship between FA and the AVLT_I_ scores for the two groups, similar to Figure 1 (see supplementary materials). However, we did not observe any significant “MD × group”, “AxD × group”, or “RD × group” interaction effects on AVLT_I_. There were no “FA, MD, AxD, or RD × group” interaction effects for the other cognitive scores.

### The association between diffusion MRI parameters and FS score

**Figure 2:**
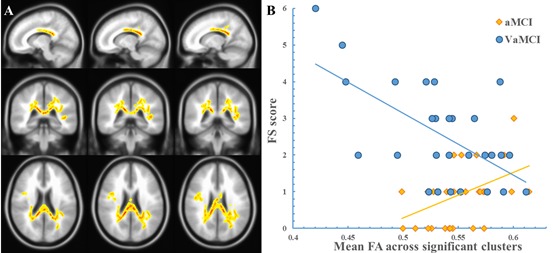
The “FA × Group” interaction effect on the FS score The clusters showing a significant “FA × group” effect are indicated in a yellow-to-red color. The color represents the F statistic for this interaction. The scatterplot was drawn using the average FA value of the cluster. Panel **A**, the region with a significant “FA × group” effect. Panel **B**, the scatterplot.

Figure 2 shows that multiple significant WM clusters exhibited an “FA × group” interaction for the FS scores, which suggests a difference in the associations of FA with the FS scores between the two groups. The significant clusters primarily covered the following areas: (1) projection fibers, such as the bilateral PCR, SCR, posterior thalamic radiation, and internal capsule; (2) association fibers, such as left cingulate gyrus, bilateral SLF; and (3) the CC (including the body and splenium). Figure 2 shows the scatterplot using the mean FA value of all significant clusters. There was a positive correlation (R = 0.55, *P* = 0.0015) between the FA and FS scores in the aMCI group, but a negative correlation in the VaMCI group (R = −0.62, *P* = 0.0011). The scatterplot of each cluster displays a very similar pattern of the relationship between FA and the FS scores for the two groups, which is similar to Figure 2 (See the supplementary materials). However, we did not observe any significant “MD × group”, “AxD × group”, or “RD × group” interaction effect on the FS score.

## DISCUSSION

The present study demonstrated that WM microstructure was not significantly different between the VaMCI and aMCI groups, which are the very early stages of two main types of clinical dementia, VD and AD. Notably, the association between WM microstructure and memory performance in this prodromal stage was significantly different between the two types of MCI. This finding provides new insights into the neuropathological progress of various dementia types.

The first major finding of our study was concerned with WM microstructure differences between the VaMCI and aMCI groups. To the best of our knowledge, our current investigation is the first study that directly compared WM microstructure between VaMCI and aMCI, which are the prodromal stages of two common dementia types. Our present negative results are compatible with previous studies that demonstrated that WM regions with FA, MD, AxD, or RD changes were similar in VaMCI and aMCI compared to healthy controls [10, 12]. One hypothesis to interpret the cognitive impairments in VaMCI patients is that brain dysfunction results from the disruption of large WM tracts[15]. Evidence of the trans-synaptic spread of tau pathology in aMCI led to the hypothesis that abnormal connectivity was a pathological mechanism for aMCI [13, 16–18]. Notably, previous studies demonstrated WM microstructure differences between VD and AD [13, 14], but our data indicated no significant differences between the two types of dementia in the pre-dementia stages. Speculatively, the divergence of WM impairments between the two types of dementia does not exist at the origin, but it gradually becomes obvious during the development and progression of dementia. This intriguing notion provides valuable insights into WM pathology and the relevant mechanisms underlying different types of dementia.

Notably, the currently used diffusion MRI parameters, including the FA, MD, AxD, and RD, are widely used to successfully detect WM microstructural changes in various brain diseases [19, 20]. However, we cannot exclude the possibility that these imaging indices exhibit a lower sensitivity for the identification of WM differences at the microscopic level. Therefore, other imaging measures for WM are encouraged to validate our currently observed negative results between the VaMCI and aMCI patients.

Notably, neither FA nor AVLT_I_ were significantly different between the two groups, but the relationship between these factors exhibited significant differences between the two groups, which were reflected by significant “FA × group” interaction effects on the AVLT_I_ in a set of clusters. These findings raise the question of whether the two different dementias affect short-term memory independently. Specifically, the AVLT_I_ scores decreased as the FA decreased in the VaMCI group. Vascular diseases likely interrupt large WM tracts of cerebral fibers directly. Therefore, the VaMCI patients with more severe WM damage likely performed worse in the AVLT_I_ and vice versa. In contrast, the aMCI patients scored higher in the AVLT_I_ as the FA decreased. The stronger WM connectivity was likely associated with a compensation mechanism that underlies the memory deficits [21]. The aMCI patients may exhibit enhanced WM connections that likely compensate for short-term memory impairments in the pre-dementia stage. This observed interaction indicates that the neuropathological differences in the early stage of dementia between different dementia subtypes may manifest in brain-cognition relationships rather than different brain phenotypes per se. The differences in brain phenotypes become obvious with the progression of dementia. Widely distributed WM impairments [9], including WM regions around the temporal lobe, cingulate and lingual gyri, CC, and bilateral posterior periventricular subcortical structures[8], are observed in VaMCI patients. However, aMCI patients exhibit WM abnormalities in the SLF, external capsule, cingulum bundle, sagittal stratum and fornix, internal capsule, corona radiate, and thalamic radiation [10, 11]. The WM regions in the current study exhibited significant interactions that were largely compatible with these previously reported regions.

The third major finding of the present study was the interaction between the FA and group on the FS score. Multiple visual rating methods for WMH/lacunar infarcts are used. The Fazekas visual rating is frequently used in clinical and research studies, and this rating method exhibits good reliability [21]. However, this manual rating system only offers a rough and global index of WM abnormality. The FS scores revealed more severe WM impairment in patients with VaMCI than aMCI, which was unlike diffusion MRI parameters. We also observed a significant group difference in the relationship between the FA and FS, which suggests that the FA-FS score relationship differed between the VaMCI and aMCI groups. A trend of more severe WM impairment in FA in the VaMCI group may be associated with an increase in the FS score. Therefore, the direction of WM abnormalities at the macrostructural level, which is represented by the FS score, and the microstructural level, which is represented by FA, were consistent in the VaMCI group. However, the correlation between FS score and FA was not that obvious in the aMCI group. This result was likely observed because FS scores are largely related to vascular risk factors, such as hypoperfusion and ischemia [22, 23], but FA is more related to neuronal degeneration caused by brain amyloid beta (Aβ) plaques or hyperphosphorylated forms of the protein tau [24, 25], which may not be notable in conventional MRI. It is difficult to determine whether their effects are the result of some unmeasured risk factors or one factor is in the etiological pathway of the other factor. Another difficulty with the FS score is that the abnormal WM signals on the T2-weighted image (T2WI) may reflect a wide spectrum of pathological changes from complete axonal degeneration to relatively benign pathology.

## LIMITATIONS AND FUTURE WORKS

First, our neuropsychological tests are limited by the primary focus on the cognitive domain of memory. Other cognitive domains, such as executive functions, should be included in the future. Second, the diagnoses for each group primarily relied on clinical diagnoses but lacked suggestive amyloid imaging or histopathological data for AD pathology in all aMCI subjects. Ongoing longitudinal follow-up studies will verify the VaMCI/aMCI subjects who eventually convert to VD or AD. Moreover, we only compared the two most common subtypes of MCI but did not include the healthy control groups and other types of pre-dementia/MCI groups, such as dementia or dementia with Lewy bodies. These pre-dementia/MCI types should be examined in future studies. Finally, our current cohort is based in a single hospital, and the sample size is relatively small. Therefore, the results require further validation by an independent dataset. Further investigation combining population-based multicenter cohorts with a large sample size should be promoted.

## MATERIALS AND METHODS

Sixty-two right-handed subjects, including twenty-eight VaMCI patients (12 men, age range: 46 ~ 77 years) and thirty-four aMCI patients (14 men, age range: 51 ~ 79 years), participated in this study. The two groups were matched in gender, age, and education. The recruited patients were outpatients who were registered at the Neurology Department of XuanWu Hospital, Capital Medical University, Beijing, China, between January 2009 and June 2015. All participants received baseline evaluations, including complete sociodemographic and clinical (cognitive, behavioral, neurological, functional, and physical) data collection. Patient histories were collected from informants, generally spouses or children. Two experienced neurologists performed the diagnoses for the two groups. The medical research ethics committee and the institutional review board of XuanWu Hospital, Capital Medical University, Beijing, China, approved this study. The study was conducted in accordance with approved guidelines, and written informed consent was obtained from all participants.

### Inclusion criteria

#### Criteria for aMCI

Diagnosis of aMCI was made based on recent international consensus criteria, which were adapted as follows [26–29]: 1) subjective cognitive complaints reported by the informant; 2) objective cognitive impairments that did not meet the Diagnostic and Statistical Manual of Mental Disorders (i.e., DSM-V) criteria for dementia; 3) normal or near-normal performance of general cognitive functioning and no or minimum impairments in daily life activities; 4) abnormal memory function, documented by extensive neuropsychological evaluation; normal general cognitive function, Clinical Dementia Rating Scale (CDR) score = 0.5 [29]; and 5) neuropsychological testing included a Hanchinski ischemic (HIS) score (HIS score ≤ 4) and MoCA (Beijing Version); cutoff points of MoCA: 13 (no formal education), 19 (1 to 6 years of education), and 24 (7 or more years of education) [30].

#### Criteria for VaMCI

Diagnosis of VaMCI depended on the following criteria [1, 12, 29, 30]: 1) subjective cognitive complaints reported by the participant or his/her caregiver; 2) insufficient cognitive impairment to meet the DSM-V criteria for dementia; 3) vascular etiology as follows: cognitive impairment due to subcortical ischemic vessel disease was defined as moderate WM changes, and/or multiple lacunar infarcts (> 2) on brain imaging; and 4) neuropsychological tests included HIS determination (HIS ≥ 7) and MoCA; cutoff points of MoCA: 13 (no formal education), 19 (1 to 6 years of education), and 24 (7 or more years of education) [30]

### Exclusion criteria

Participants were excluded if they exhibited any of the following conditions: 1) psychiatric disease (e.g., depression, Hamilton depression rating scale > 20, Center for Epidemiologic Studies Depression Scale > 21), systemic disease or other neurological disorder; 2) visual or auditory abnormalities that made clinical assessments infeasible; 3) alcohol or drug abuse; 4) insufficient Mandarin language abilities to complete the assessment; 5) MRI contraindications, such as claustrophobia; or 6) marked head-motion according to the MRI image.

The exclusive criteria for VaMCI also included the following conditions: signs of large vascular disease, such as cortical, and/or cortico-subcortical, or non-lacunar territorial infarcts and watershed infarcts or hemorrhages.

### Neuropsychological evaluations

All participants underwent a battery of neuropsychological tests to assess general mental status and other cognitive domains. These tests included CDR scale [28, 29], MMSE [31], MoCA [30], and AVLT [32]. Two attending neurologists performed all evaluations.

### Scanning parameters

The imaging scans were acquired on a 3.0 Tesla Siemens Tim Trio MRI scanner at the XuanWu Hospital. T2WIs were acquired using the following sequence: repetition time (TR) = 4040 ms; echo time (TE) = 84.0 ms; flip angle = 160°; field of view = 240 mm × 240 mm; matrix = 186 × 320; 20 slices; and slice thickness = 5.0 mm. Diffusion MRI imaging sequence: An echo-planar imaging sequence with one zero-weighted image (b = 0 s/mm^2^) and 30 diffusion sensitizing orientations (b = 1000 s/mm^2^) was used with the following parameters: slice thickness = 2 mm; 90 slices; TR = 11000 ms; TE = 98 ms; voxel size = 2 mm isotropic; flip angle = 90°; acquisition matrix = 128 mm × 116 mm; and number of averages = 3.

### WMH/lacunar infarct rating

WM lesion severity was rated using a modified FS score [33]. A single experienced investigator analyzed the T2WI scans of each participant and graded WMH signal surrounding the ventricles and deep WM according to FS. Periventricular WMH (PWMH) was graded as 0 = absence, 1 = “caps” or pencil-thin lining, 2 = smooth “halo”, or 3 = irregular periventricular hyperintensities extending into the deep WM. Separate deep WMH signals (DWMH) were rated as 0 = absence, 1 = punctate foci, 2 = beginning confluence of foci, or 3 = large confluent areas. The rater was blinded to the clinical data of participants. The total WMH score was calculated as the sum of the PWMH and DWMH scores [33].

### Diffusion MRI preprocessing

Diffusion-weighted images were processed using PANDA [34], which is an automatic processing pipeline based on Matlab and FSL [35]. The images were skull-striped. Head movement and eddy current corrections were performed. FA images were calculated. Individual FA images for each subject were co-registered to the Montreal Neurological Institute (MNI) space. These aligned images were averaged and thresholded at 0.2 to extract a mean FA skeleton onto which individual FA data were projected. The MD, AxD, and RD were also calculated and projected onto the same skeleton.

### Statistical analysis

Statistics for demographics were calculated using Chi-squared and Student's t tests, when appropriate. Statistical comparisons of the spatial distributions of abnormal WMH/lacunar infarct regions were performed using a Chi-squared test to evaluate whether there were gross differences in WMH/lacunar infarcts between the two groups.

Statistical analyses were performed on the FA, MD, AxD, and RD data using the TBSS method [36]. The TBSS method applies statistical analyses throughout the entire WM skeleton that was extracted from the mean FA map in the MNI space (see supplementary materials). This method does not require spatial smoothing and provides a more reliable alignment of the WM tracts and overcomes prior knowledge limitations of the region-of-interest approach while preserving regional information and specificity [36]. The FA, MD, AxD, and RD values for each WM skeleton voxel were statistically compared between the aMCI and VaMCI groups using 2-sample t tests (two-tailed). Age, gender, and education were included as covariates. We used a general linear model (GLM) to determine whether there was a significant “FA, MD, AxD, or RD × group” interaction on cognitive measures or a significant “FA × group” interaction on the FS score. The cognition measures included the MMSE, MoCa and three subtests of the AVLT. The threshold-free cluster enhancement (TFCE) approach with 5000 permutations was used to correct for multiple comparison [37], and FWE corrected P < 0.05 was considered significant.

## SUPPLEMENTARY MATERIALS


