# Supplementary material for: How does white matter microstructure differ between the vascular and amnestic mild cognitive impairment?
Source: Oncotarget. 2016 Dec 15;8(1):42–50. doi: 10.18632/oncotarget.13960 (PMC5352131; doi:10.18632/oncotarget.13960)
Supplement: Supplementary file 1 [file oncotarget-08-42-s001.pdf]

How does white matter microstructure differ between the vascular and amnestic mild cognitive impairment?

Supplementary Materials

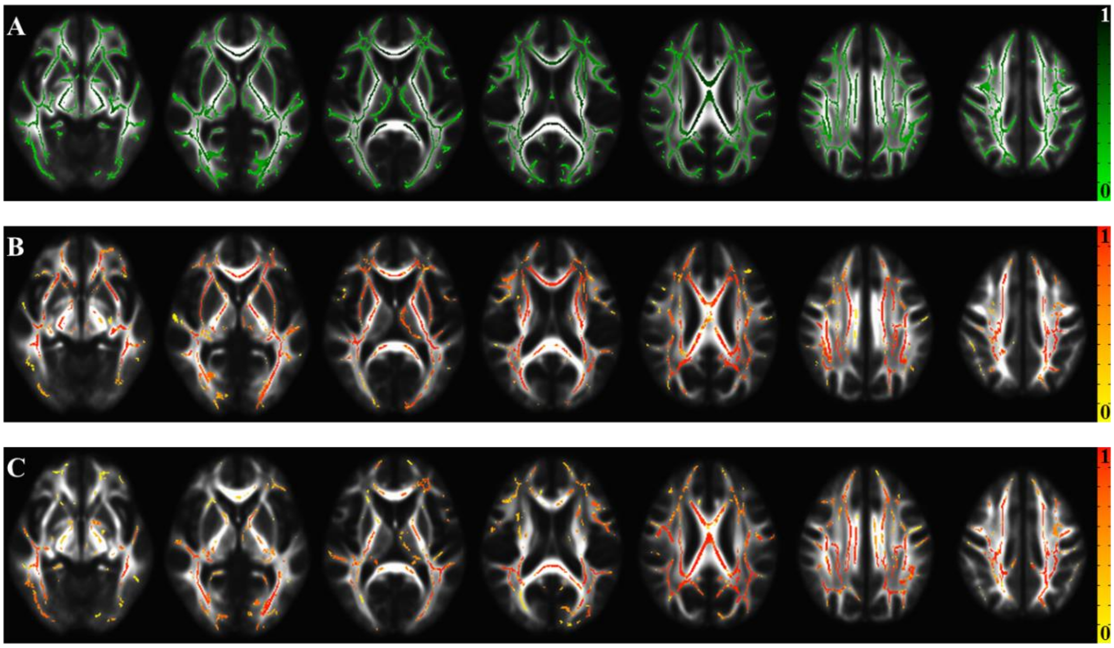

Figure S1. The mean FA skeleton and corrected P value map. Panel A shows the FA skeleton for the TBSS analysis. Panel B shows (1 - P value) map of "FA × Group" interaction effect on $AVLT_I$ after TFCE correction. Panel C shows (1 - P value) map of "FA × Group" interaction effect on the FS score after TFCE correction.

Text S1:

The effect of the “FA × Group” interaction on the $AVLT_I$.

There were multiple significant clusters in the “FA × group” effect on the $AVLT_I$. We created a scatterplot for each of the clusters and found that these clusters exhibited a very similar pattern for the relationship between the FA and the $AVLT_I$ in the two groups. Here, we show the region and scatterplot of the first three clusters.

First cluster with 524 voxels:

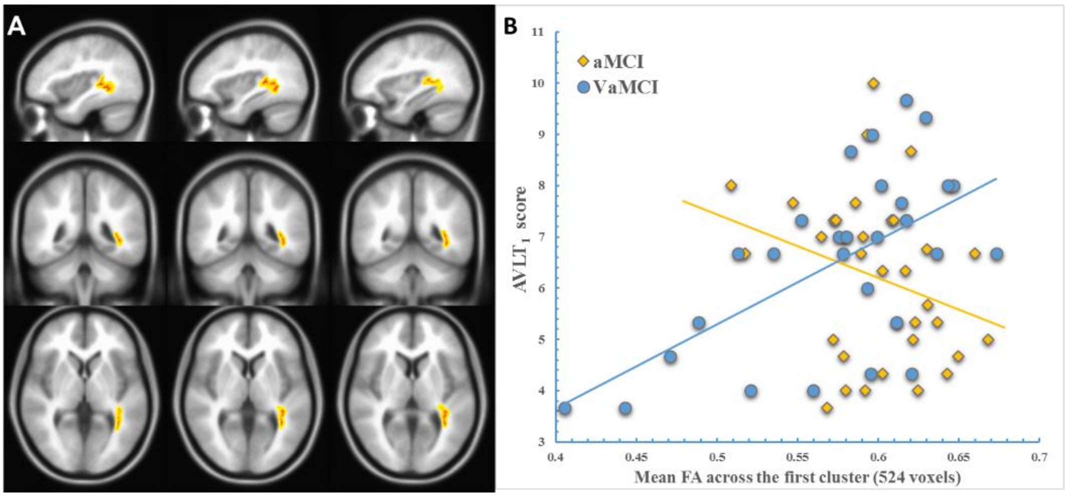

Figure S2.1 The effect of the “FA × Group” interaction of the first cluster with 524 voxels on the $AVLT_I$. The clusters showing a significant “FA × group” effect are indicated in a yellow-to-red color. The color represents the F statistic for this interaction. The scatterplot was drawn using the average FA value of the cluster. Panel A, region with a significant “FA × group” effect. Panel B, the scatterplot.

Second cluster with 507 voxels:

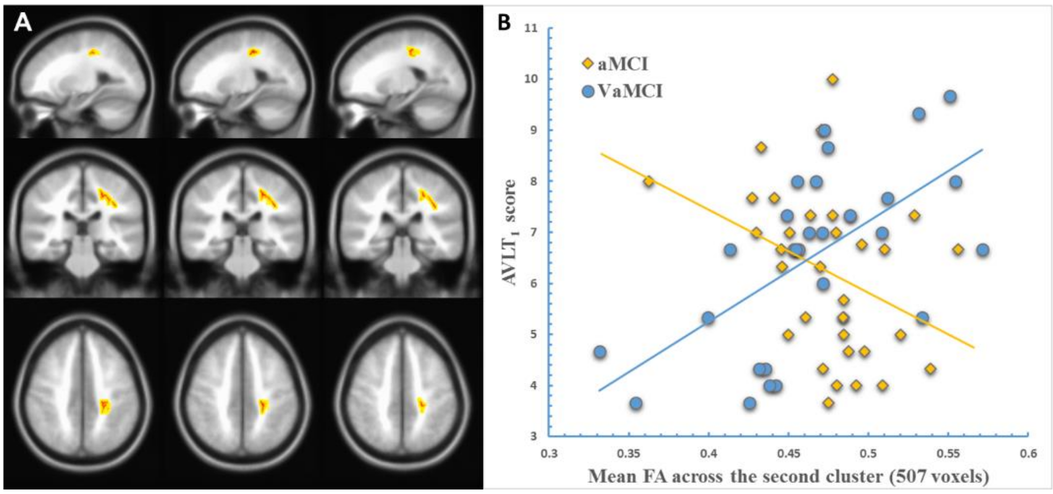

Figure S2.2 The effect of the "FA × Group" interaction of the second cluster with 507 voxels on the AVLT$_{I}$. The clusters showing a significant "FA × group" effect are indicated in a yellow-to-red color. The color represents the F statistic for this interaction. The scatterplot was drawn using the average FA value of the cluster. Panel A, region with a significant "FA × group" effect. Panel B, the scatterplot.

Third cluster with 287 voxels

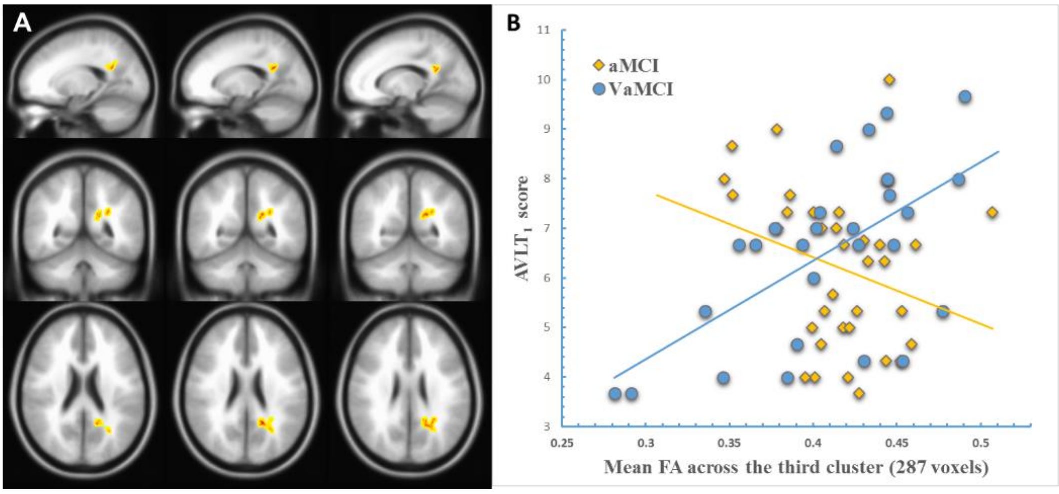

Figure S2.3 The effect of the "FA × Group" interaction of the third cluster with 287 voxels on the AVLT$_{I}$. The clusters showing a significant "FA × group" effect are indicated in a yellow-to-red color. The color represents the F statistic for this interaction. The scatterplot was drawn using the average FA value of the cluster. Panel A, region with a significant "FA × group" effect. Panel B, the scatterplot.

Text S2:

The effect of the “FS × Group” interaction on the FS score:

There were also multiple significant clusters for the “FA × group” effect on the FS scores. Each of the clusters showed a very similar pattern for the relationship between the FA and the Fs score in the two groups. We also show the region and scatterplot of the first three clusters.

First cluster with 953 voxels:

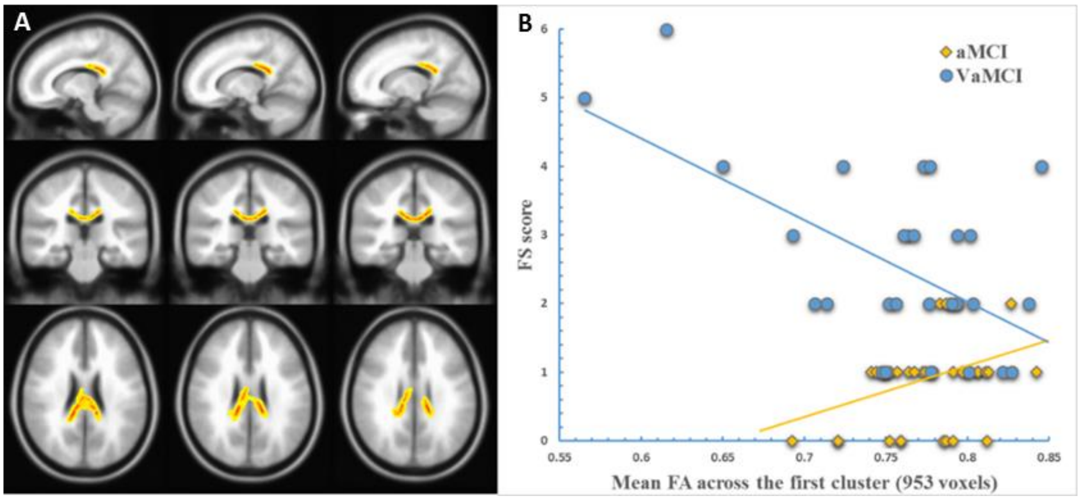

Figure S3.1 The effect of the “FS × Group” interaction of first cluster with 953 voxels on the FS score. The clusters showing a significant “FS × group” effect are indicated in a yellow-to-red color. The color represents the F statistic for this interaction. The scatterplot was drawn using the average FA value of the cluster. Panel A, the region with a significant “FS × group” effect. Panel B, the scatterplot.

Second cluster with 362 voxels:

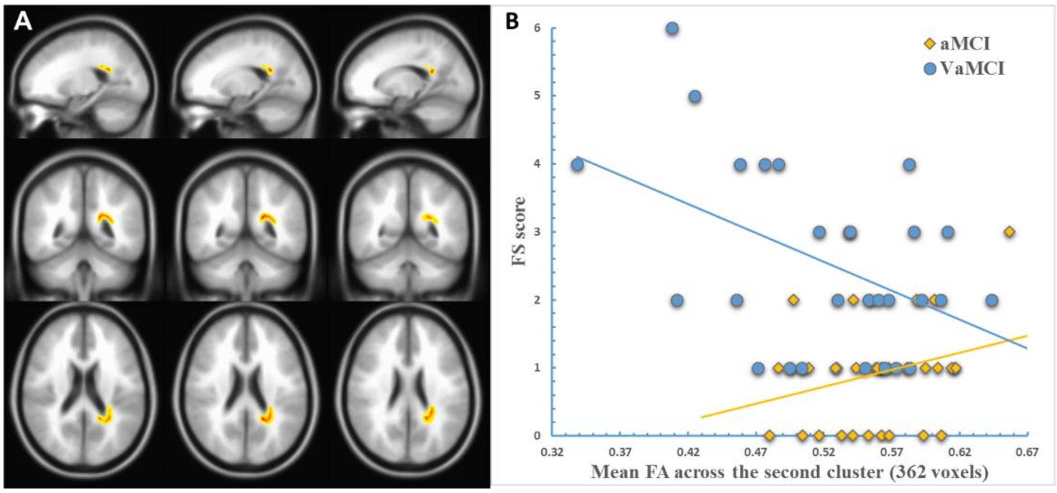

Figure S3.2 The effect of the "FS × Group" interaction of the second cluster with 362 voxels on the FS score. The clusters showing a significant "FS × group" effect are indicated in a yellow-to-red color. The color represents the F statistic for this interaction. The scatterplot was drawn using the average FA value of the cluster. Panel A, the region with a significant "FS × group" effect. Panel B, the scatterplot.

Third cluster with 358 voxels:

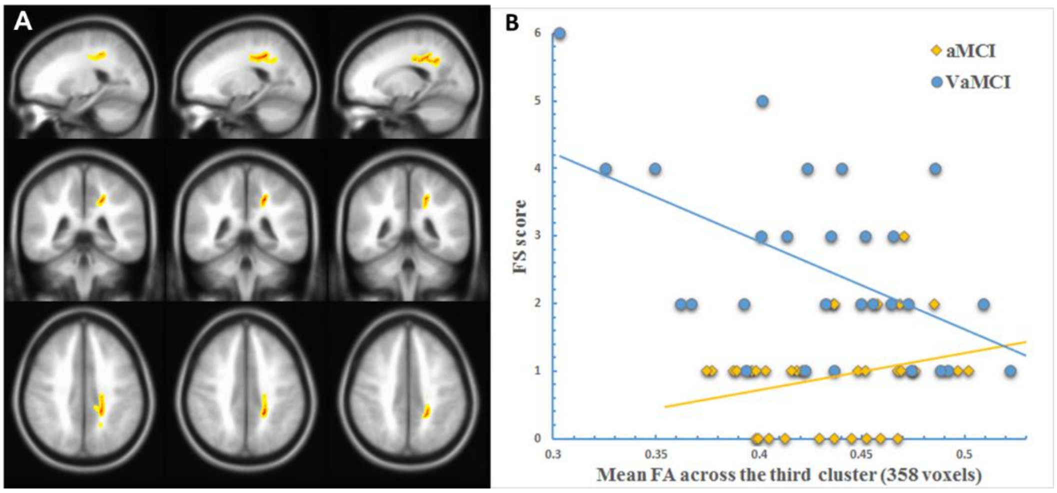

Figure S3.3 The effect of the "FS × Group" interaction of the third cluster with 358 voxels on the FS score. The clusters showing a significant "FS × group" effect are indicated in a yellow-to-red color. The color represents the F statistic for this interaction. The scatterplot was drawn using the average FA value of the cluster. Panel A, the region with a significant "FS × group" effect. Panel B, the scatterplot.
